# Utilization of Emergency Department Services by the Bedouin Population in Southern Israel

**DOI:** 10.1100/tsw.2007.35

**Published:** 2007-03-02

**Authors:** Arnon Dov Cohen, Jacob Dreiher, Amir Sharf, Daniel Aharon Vardy

**Affiliations:** ^1^ Clalit Health Services, Southern District, Israel; ^2^ Siaal Research Center for Family Medicine and Primary Care, Faculty of Health Sciences, Ben-Gurion University of the Negev, POB 653, Beer-Sheva 84150, Israel

**Keywords:** Bedouins, Israel, healthcare utilization, Arabs, primary care, emergency medicine

## Abstract

Excessive use of the emergency department (ED) is associated with increased costs and workload in the ED, patients' inconvenience and disruption of the continuity of care. The study's goal was to describe trends in ED utilization among Bedouins living in southern Israel. A retrospective cross-sectional study was conducted in primary care clinics in southern Israel. Patients included Bedouin and Jewish patients insured by Clalit Health Services. Data was retrieved from a central database. The number of visits to the ED and age-adjusted rates of ED visits during 2000-2003 were determined in the Bedouin vs. Jewish population. All visits that ended in hospitalization were excluded. Data was stratified according to patients' residence (semi-nomadic vs. urban Bedouins) and referral origin. Age-adjusted rates of ED visits decreased from 42.9/1000 patients/month in 2000 to 38.3/1000 patients/month in 2003. There were more ED visits in the Bedouin as compared to Jewish population (38.3/1000 vs. 21.8/1000 patients/month). The decrease in ED utilization was more prominent among adult semi-nomadic Bedouins (from 60.8/1000 to 40.3/1000 patients/month). The proportion of referrals by the family physician to ED significantly decreased (among urban Bedouins: from 54.3% to 43.2%, p<0.001; among semi-nomadic Bedouins: from 53.9% to 39.9%, p<0.001), while the proportion of selfreferrals and referrals from physicians other than the family physician increased. A decrease in ED utilization by the Bedouin population during the last years was demonstrated. Utilization of ED services is still increased as compared to the non-Bedouin population. Interventions to control excessive use of ED services in the Bedouin population are currently underway.

## INTRODUCTION

Emergency departments (EDs) in Israeli hospitals deal with an increasing number of visits. In the past, several studies suggested that having a regular and consistent source of healthcare increases patient and provider satisfaction, decreases hospitalization rate, and lowers costs. Thus, excessive use of ED is associated with increased costs, increased workload in the EDs and patients' inconvenience[1-10].

The Bedouins in the Negev comprises about 150,000 people. This is a population in transition from nomadic to urban form of life. About 80% of the population lives in permanent settlements and the other 20% hold a traditional, semi-nomadic lifestyle in small groups of tents or shacks without proper infrastructure, electricity or running water. The transition from a traditional to a western lifestyle, characterized by changes in dietary habits[11] and a reduction in physical activity is associated with substantial changes in morbidity patterns, such as an increase in the rates of hypertension, diabetes mellitus[11,12,13], home accidents[14] and asthma[15].

The tertiary regional hospital, Soroka University Medical Center, located in the central town of Beer-Sheva, serves as the major referral center for the vast majority of the Bedouin population in the Negev. Public transportation is limited to the main towns, and many semi-nomadic Bedouin live in remote locations with no paved roads, making unnecessary use of ED services a burden on the population. A visit to the ED bares different costs to the patient depending on the source of referral (physician-initiated vs. self-referral). In a population of a low socioeconomic status such as the Bedouins, even minor differences in costs are significant. From the healthcare purchaser's point of view, efficient use of the ED remains an important challenge.

The aim of the current study is to describe trends in the utilization of ED services by Bedouins living in the Negev region in southern Israel.

## METHODS

Clalit Health Services is the largest health maintaining organization in Israel. In the southern district of Israel, Clalit Health Services covers a population of approximately 470,000 enrollees. The Bedouin population of the Negev comprises about 150,000 people. The vast majority of the Bedouin population(about 120,000) is treated by the Southern Negev Administration, which is part of the southern district of Clalit Health Services. About 80% of these live in settlements, while the rest are semi-nomadic. In the recent decade, Clalit Health Services has built numerous clinics for seminomadic Bedouins, and expanded the existing clinics in Bedouin settlements. It also computerized its medical record, and created a database, which may be used for assessment of health utilization using data mining techniques. Previous studied have described the utilization of health services[2,16,17] and health intervention programs in the Bedouin population[14,15].

The Clalit Health Services' database was reviewed to identify all the visits made by Bedouin patients to ED. Included in the study were visits to the district hospital (Soroka University Medical Center), which ended in the patient's discharge (visits in which the patient was hospitalized were excluded). The following variables were extracted from the database: date of the visit, patients’ address, age, referral origin, and section of ED. Data was stratified according to the place of residence (urban vs. semi-nomadic), patients' age, ED section and referral origin. No data were available regarding the reasons for referral. The rate of visits to the ED was age-adjusted using the direct method with the total Clalit health services' enrollees as the reference population. Chi-square test was used to compare categorical parameters between the groups.

## RESULTS

By searching the computerized database, a total of between 57,710 visits to the ED in 2000 (out of 153,323 enrollees) and 56,025 visits in 2003 (out of 175,546 enrollees) were identified (all ending in the patient's discharge). Among Bedouins, the age-adjusted rate of ED visits decreased from 42.9/1000 patients/month in 2000 to 38.3/1000 patients/month in 2003. The number of age-adjusted ED visits was higher in the Bedouin population as compared to the non-Bedouin population (38.3/1000 patients/month vs. 21.8/1000 patients/month, respectively). Table 1 displays trends in the number of ED visits by urban and semi-nomadic Bedouins between 2000 and 2003.

The number of visits to the ED varied according to the patients' age. The decrease in ED utilization was more prominent in semi-nomadic patients above 15 years as compared with urban patients (60.8/1000 patients/month in 2000 vs. 40.3/1000 patients/month in 2003, respectively). The numbers of ED visits by Bedouin patients in selected age groups are shown in Table 2.

The proportion of referrals by the family physician to the ED (of the total number of visits) significantly decreased from 2000 to 2003 (Urban: 54.3% and 43.2%, respectively, p-value<0.001; semi-nomadic: 53.9% and 39.9%, respectively, p-value<0.001), in conjunction with an increase in the proportion of self referrals and referrals from physicians other than the family physicians (Table 3).

In both the Bedouin and non-Bedouin populations, there was a decrease in the proportion of ED visits in the surgical section of the ED, which occurred in conjunction with an increase in the proportion of visits to the pediatrics and obstetrics and gynecology sections of the ED (Table 4).

## DISCUSSION

In the current study, trends in ED use by the Bedouin population residing in the Negev region were analyzed. Age-adjusted ED utilization rates significantly decreased within a 3-year period. In our opinion, the decrease in ED utilization reflects the improvement of primary care services for the Bedouin population during the years 2000-2003. In these years Clalit Health Services opened new clinics in Bedouin towns and tribes and allocated board-certified family physicians to remote clinics. The process of decentralization of the medical management of large clinics, which made medical directors in each clinic responsible for balancing their own clinic's budget and quality of service outcomes, further facilitated the process of lowering excessive use of ED services.

Current rates of ED utilization rates are still high as compared to the non-Bedouin population. Several explanations for the excessive use of ED services can be suggested.

**TABLE 1**
**Trends in rates of ED visits by Bedouin patients, 2000-2003**

| Year | Non-Bedouin | | | Bedouin (All) | | | Urban Bedouins | | | Semi-nomadic Bedouins | | |
|---|---|---|---|---|---|---|---|---|---|---|---|---|
| | N | ED Visits | Rate | N | ED Visits | Rate | N | ED Visits | Rate | N | ED Visits | Rate |
| 2000 | 55,862 | 18,258 | 26.5 | 97,461 | 39,452 | 42.9 | 83,772 | 32,332 | 42.5 | 13,689 | 7,120 | 44.7 |
| 2001 | 56,706 | 17,346 | 24.6 | 104,211 | 41,418 | 42.8 | 88.276 | 33,613 | 42.1 | 15,808 | 7,805 | 46.6 |
| 2002 | 56,924 | 15,645 | 22.0 | 112,122 | 38,652 | 37.8 | 92,647 | 31,685 | 37.9 | 19,263 | 6,967 | 37.6 |
| 2003 | 59,396 | 16,265 | 21.8 | 116,150 | 39,760 | 38.3 | 94,043 | 32,113 | 38.0 | 22,107 | 7,647 | 39.5 |
| P value | <0.001 | | | <0.001 | | | <0.001 | | | <0.001 | | |

N represents the number of enrollees. ED (emergency department) visits is the total number of visits per year.
"Rate" is the age-adjusted rate of ED visits per 1000 enrollees, per month.

## ED As A Surrogate for Laboratory, Imaging or Consultation Services

Some patients (and physicians) use the ED as a surrogate for laboratory, imaging or consultation services despite the fact that imaging and consultation services were introduced in Bedouin clinics (both in settlements and tribes). In previous studies, we have described a similar trend among soldiers of the Israel Defense Force who use the ED in Soroka University Medical Center as a surrogate for ambulatory services[9,10].

It should be noted that in all the Bedouin clinics, laboratory services are available through a network of transportation to the central laboratory in Beer-Sheva.

**TABLE 2**
**Rates of ED visits by Bedouin patients, in selected age groups, in 2000 and 2003**

| Age-group | Year | Non-Bedouin | | | Bedouin (All) | | | Urban Bedouins | | | Semi-nomadic Bedouins | | |
|---|---|---|---|---|---|---|---|---|---|---|---|---|---|
| | | N | ED Visits | Rate | N | ED Visits | Rate | N | ED Visits | Rate | N | ED Visits | Rate |
| 0-4 y | 2000 | 5,154 | 1,589 | 25.7 | 21,719 | 8,800 | 33.8 | 18,648 | 7,186 | 32.1 | 3,071 | 1,614 | 43.8 |
| | 2003 | 5,716 | 1,385 | 20.2 | 104,211 | 41,418 | 30.1 | 17,558 | 6,318 | 30.0 | 4,242 | 1,555 | 30.5 |
| | P value | | <0.001 | | | <0.001 | | | <0.001 | | | <0.001 | |
| 5-14 y | 2000 | 11,378 | 1,632 | 12.0 | 30,572 | 5,088 | 13.9 | 26,207 | 4,147 | 13.2 | 4,365 | 941 | 18.0 |
| | 2003 | 10,913 | 1,379 | 10.5 | 36,493 | 5,050 | 11.5 | 29,448 | 4,193 | 11.9 | 7,045 | 857 | 10.1 |
| | P value | | <0.001 | | | <0.001 | | | <0.001 | | | <0.001 | |
| 15y + | 2000 | 39,330 | 15,037 | 31.8 | 45,170 | 25,564 | 47.1 | 38,917 | 20,999 | 45.0 | 6,253 | 4,565 | 60.8 |
| | 2003 | 42,767 | 13.501 | 26.3 | 57,857 | 26,837 | 38.7 | 47,037 | 21,602 | 38.3 | 10,820 | 5,235 | 40.3 |
| | P value | | <0.001 | | | <0.001 | | | <0.001 | | | <0.001 | |

N represents the number of enrollees. ED (emergency department) visits is the total number of visits per year.
"Rate" is the age-adjusted rate of ED visits per 1000 enrollees, per month.

**TABLE 3**
**Referral origin for ED visits in the Bedouin population, in 2000 and 2003**

| Referral origin | Non-Bedouin | | Urban Bedouins | | Semi-nomadic Bedouins | |
|---|---|---|---|---|---|---|
| | 2000 | 2003 | 2000 | 2003 | 2000 | 2003 |
| | N=18,258 | N=16,265 | N=32,332 | N=32,113 | N=7,120 | N=7,647 |
| Family physician | 10,266 (56.2%) | 7,677 (47.2%) | 17,550 (54.3%) | 13,885 (43.2%) | 3,841 (53.9%) | 3,048 (39.9%) |
| Other physician | 5,201 (28.5%) | 5,389 (33.1%) | 8,008 (24.8%) | 10,092 (31.4%) | 1,389 (19.6%) | 2,306 (30.1%) |
| Self referral | 2,791 (15.3%) | 3,199 (19.7%) | 6,774 (21.0%) | 8,136 (25.3%) | 1,890 (26.5%) | 2,293 (30.0%) |
| P value | <0.001 | | <0.001 | | <0.001 | |

The numbers in the table represent the percentage of the referral origin, from the total number of ED visits.

## The Erroneous Concept That Hospital Doctors are "Better"

The notion that hospital doctors are "better" than community doctors was prominent among the Bedouin population over the last decades. Despite the fact that board-certified family physicians and pediatricians are constantly assigned to work in Bedouin clinics, the advantages of high-quality community care is spreading rather slowly in the Bedouin community.

**TABLE 4**
**Proportion of ED section of all visits in 2000 and 2003**

| ED section | Non-Bedouins | | Bedouin | |
|---|---|---|---|---|
| | 2000 | 2003 | 2000 | 2003 |
| | N=18,258 | N=16,265 | N=39,452 | N=39,760 |
| Pediatrics | 2,224 (12.2%) | 2,454 (15.1%) | 9,223 (23.4%) | 10,656 (26.8%) |
| Surgery | 9,230 (50.6%) | 6,987 (43.0%) | 17,459 (44.3%) | 14,909 (37.5%) |
| Obstetrics / gynecology | 2,261 (12.4%) | 2,551 (15.7%) | 6,740 (17.1%) | 8,032 (20.2%) |
| Internal medicine | 4,543 (24.9%) | 4,273 (26.3%) | 6,030 (15.3%) | 6,163 (15.5%) |
| P value | p<0.001 | | p<0.001 | |

The numbers in the table represent the percentage of the ED section, from the total number of ED visits.

## Referrals To The ED by Private Physicians

A third reason for the elevated use of ED services may be due to the work of private physicians in the Bedouin population. These physicians are in many cases general practitioners who are not board-certified and work from their home. These private physicians may refer patients to the ED (usually after regular clinic hours) due to their lack of medical knowledge or lack of familiarity with the medical system in the Negev. They might be less concerned with the economical consequences of excessive ED use on the health maintaining organizations, as compared with physicians who are employed by the health maintaining organizations.

The decrease in ED utilization was more prominent among semi-nomadic Bedouins as compared with urban Bedouins. This observation reflects the opening of clinics in Bedouin tribes, providing the population with an access to board-certified family physician that have the knowledge and responsibility to treat the majority of medical problems, without the need for referral to the ED.

In summary, despite substantial decrease in ED utilization by the Bedouin community, ED utilization is still high as compared to the non-Bedouin population. Focused interventions for control of the excess use of ED service in the Bedouin population are currently performed by Clalit Health Services. These efforts include opening of new clinics in the Bedouin towns and tribes and allocation of board-certified primary care physicians and consultants to these clinics. We believe that in future years the quality of medical care in Bedouin primary care clinics will increase, with a further decrease in the need for the use of ED services.
